# Microbial Valorization of Lignin to Bioplastic by Genome-Reduced *Pseudomonas putida*

**DOI:** 10.3389/fmicb.2022.923664

**Published:** 2022-05-30

**Authors:** Qiu-Jin Zong, Tao Xu, He Liu, Li Xu, Ren-Kuan Zhang, Bing-Zhi Li, Zhi-Hua Liu, Ying-Jin Yuan

**Affiliations:** Frontiers Science Center for Synthetic Biology and Key Laboratory of Systems Bioengineering (Ministry of Education), School of Chemical Engineering and Technology, Tianjin University, Tianjin, China

**Keywords:** biological lignin valorization, fed-batch strategy, alkaline pretreated liquor, polyhydroxyalkanoates, lignin characterization

## Abstract

As the most abundant natural aromatic resource, lignin valorization will contribute to a feasible biobased economy. Recently, biological lignin valorization has been advocated since ligninolytic microbes possess proficient funneling pathways of lignin to valuable products. In the present study, the potential to convert an actual lignin stream into polyhydroxyalkanoates (PHAs) had been evaluated using ligninolytic genome-reduced *Pseudomonas putida*. The results showed that the genome-reduced *P. putida* can grow well on an actual lignin stream to successfully yield a high PHA content and titer. The designed fermentation strategy almost eliminated the substrate effects of lignin on PHA accumulation. Employing a fed-batch strategy produced the comparable PHA contents and titers of 0.35 g/g dried cells and 1.4 g/L, respectively. The molecular mechanism analysis unveiled that *P. putida* consumed more small and hydrophilic lignin molecules to stimulate cell growth and PHA accumulation. Overall, the genome-reduced *P. putida* exhibited a superior capacity of lignin bioconversion and promote PHA accumulation, providing a promising route for sustainable lignin valorization.

## Introduction

Given concerns over fossil resource shortages and climate change, the development of alternative and sustainable resources is urgently required for the production of biofuels, chemicals, and materials (Ragauskas et al., 2014; Erickson et al., 2022). Lignin is one of three main components in lignocellulosic biomass and the most abundant renewable aromatic resource in nature, which is considered a promising alternative for the production of valuable products (Ragauskas et al., 2014; Liu et al., 2019b). Large amounts of lignin are being generated from the biorefinery and the paper and pulp industry; however, lignin remains an underutilized solid residue or is only burnt for heat and power generation. Currently, it is of great interest to realize the potential value of lignin since lignin valorization not only produces valuable commodities but also contributes to a low-carbon economy (Abu-Omar et al., 2021; Liu et al., 2021).

**Figure 1:**
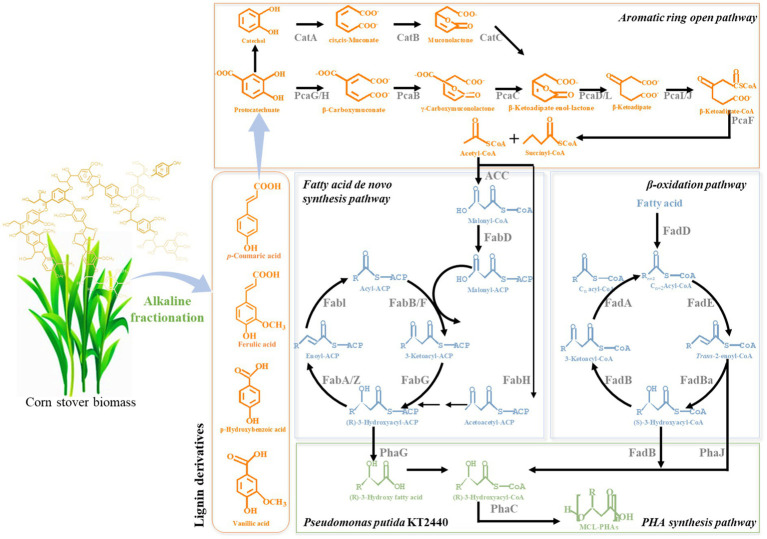
Biological lignin valorization for the production of eco-friendly bioplastics from alkaline fractionated lignin using ligninolytic genome-reduced *Pseudomonas putida* strain.

Recently, biological lignin valorization has shown the promising potential, as various microorganisms in nature have evolved metabolic pathways of lignin and aromatics (Figure 1; Beckham et al., 2016; Liu et al., 2019b; Zhang et al., 2019). For example, ligninolytic microbe, *Pseudomonas putida*, can metabolize heterogonous aromatics derived from lignin *via* a “biological funnel” to yield central aromatic intermediates, protocatechuate, or catechol (Linger et al., 2014; Zhang et al., 2021b). These intermediates can be further catabolized *via* the *β*-ketoadipate pathway to produce acetyl-CoA and facilitate the synthesis of polyhydroxyalkanoates (PHAs; Gonçalves et al., 2020). PHAs comprise a group of natural biopolyesters, which have a potential to serve as an alternative to fossil-based plastics (Borrero-de Acuña et al., 2021; Zhang et al., 2021c). They are also biodegradable and biocompatible polymers for medical and material applications (Raza et al., 2018; Estevez-Alonso et al., 2021). Despite the potential, PHAs have not yet been commercialized at an industrial scale due to the high cost of raw materials, contributing to approximately 50% of the total production cost (Kumar et al., 2017). Biological valorization of inexpensive lignin to eco-friendly bioplastic could improve economic viability and environmental sustainability. To realize the bioconversion potential of lignin to PHAs, efforts are still needed to enhance the capacity of the ligninolytic strains, exploit feasible fermentation strategies, and improve the bioavailability of lignin.

Moderate genome reduction of unnecessary cellular processes is a feasible and emerging strategy to construct a microbial chassis with optimized cellular metabolic pathways, enhanced substrate utilization capacity, and improved controllability of product synthesis (Kuhner et al., 2009; Choe et al., 2019). A series of genome-reduced *P. putida* strains greatly improved the utilization capabilities of glucose substrate, and significantly increased PHA yield and cell growth (Liang et al., 2020; Weimer et al., 2020). Most importantly, *P. putida*, which harbors reduced genomes, has excellent genetic stability and elevated resistance to oxidative stress. Genome-reduced *P. putida* could be act as a promising chassis to construct cell factories of lignin bioconversion. However, as a ligninolytic strain, genome-reduced *P. putida* has not been evaluated to convert lignin into PHAs. Understanding the cell growth and PHA accumulation behaviors of genome-reduced *P. putida* is necessary, as it will guide strain design to promote lignin bioconversion.

The original aromatic structure and the macromolecular properties of lignin are the most important factors impacting lignin bioavailability and bioprocessing. Although the most recent works on metabolic engineering have successfully enhanced PHA production from lignin residues or aromatic compounds, the PHA titer need to be further improved to make a feasible bioconversion. PHA accumulation in microbes is sensitive to the properties of lignin and a number of environmental factors. Lignin in nature is a hydrophobic biopolymer that is synthesized from *p*-coumaryl, coniferyl, and sinapyl alcohol *via* a variety of carbon–carbon or carbon–oxygen linkages (Guo et al., 2021). To make bioconversion reliable, it is thus crucial to deconstruct the lignin polymer and improve its solubility and bioavailability by yielding small molecules and aromatics (Liu et al., 2017). Furthermore, fermentation mode options are also crucial to lignin bioconversion and PHA accumulation. For example, optimizing medium components is of great importance (Singh et al., 2017), as PHAs are generally formed in microbes with an excess carbon source and limiting nitrogen conditions. Although a high product titer requires fermentation at a high substrate concentration, increases in lignin concentration may adversely influence cell growth due to inhibitory effects. Few studies have described the exploitation of fermentation modes to improve lignin bioconversion and PHA accumulation using genome-reduced *P. putida*.

The aim of this work was to improve lignin valorization by converting an actual lignin stream to PHAs using genome-reduced *P. putida*. Alkaline fractionation had been employed to improve the bioavailability of lignin to microbes. The ligninolytic capacity and PHA accumulation of genome-reduced *P. putida* strains were investigated using an actual lignin stream. The effects of the carbon to nitrogen ratio, pH control strategy, harvesting time, and lignin concentration on PHA accumulation were then systematically evaluated. After that, various fermentation strategies were designed to promote the PHA accumulation. Genome-reduced *P. putida* together with fed-batch mode design could facilitate lignin bioconversion and PHA accumulation.

## Materials and Methods

### Alkaline Fractionation of Lignin From Corn Stover

Corn stover biomass was harvested from the suburbs of Tianjin, China. Corn stover was water-washed to remove ash, extractives, and other impurities and then dried to a moisture weight of 5%–10%. The dried corn stover was milled and sieved to collect the fractions between 20 and 80 mesh for further use.

For lignin preparation, alkaline fractionation using sodium hydroxide was conducted to fractionate lignin from corn stover in a sealed stainless-steel reactor. Mixtures of corn stover, sodium hydroxide, and water were prepared at a 10% solid loading with 100 mg NaOH/g corn stover. The mixture was loaded into the reactor and heated to 130°C for 30 min. After that, alkaline pretreated liquor (APL) was separated by removing the solid residue using the filtration method. The liquid fraction was centrifuged at 12,000 rpm for 10 min to separate suspended fine particles in APL. APL mainly containing soluble lignin was collected and used as carbon source in fermentation. Calcium lignosulfonate (CLS) powder was purchased from Tianjin Damao Chemical Reagent Factory, Tianjin, China. The composition of APL and CLS was shown in Table 1.

**Table 1:** The composition of alkaline pretreated liquor (APL) from corn stover biomass and calcium lignosulfonate (CLS).

Composition		APL (g/L)	CLS (g/g)
Lignin	Soluble lignin	15.8	0.32
Glucan	Glucose	-	0.01
	Oligo-glucose	0.7	0.04
Xylan	Xylose	-	0.02
	Oligo-xylose	8.2	0.18
Arabinan	Oligo-arabinose	3.2	-
Ash		15.1	0.14
Others		10.2	0.28

### Strain and Seed Culture Preparation

*Pseudomonas putida* strains were kindly provided by Chao Yang from the Key Laboratory of Molecular Microbiology and Technology, Ministry of Education, Nankai University, Tianjin, China. The genome-reduced *P. putida* strains were named KTU, KTU-U3, and KTU-U13, according to the deleted regions of genomic islands (GIs; Liang et al., 2020).

About 1 L seed medium was prepared with 100 ml 10 × M9, 10 ml 12 g/L MgSO_4_·H_2_O, 100 ml 200 g/L glucose, and 100 μl trace element solution. Phosphate buffer was added to the solution, and the medium was then finalized to 1.0 L. Trace element solution was prepared with the procedures described in previous study (Liu et al., 2018). NH_4_Cl was used as a nitrogen source by diluting to the desired concentration.

For seed culture, the strain was cultured on Luria-Bertani (LB) medium plates. A single colony was selected and inoculated into 5 ml LB medium and grown at 30°C for 12 h with a shaking speed of 220 rpm. About 1 ml of liquid medium was then transferred into 50 ml of medium for seed culture. The seed cells were collected at the late exponential growth phase with an OD_600_ of approximately 7. The harvested cells were washed with sterile phosphate-buffered saline (PBS) and then transferred into lignin medium for PHA fermentation.

### PHA Fermentation Using Lignin as Carbon Source

Alkaline pretreated liquor fractioned from corn stover biomass was used as carbon source for KTU, KTU-U3, and KTU-U13 strains to produce PHAs. For medium preparation, APL was adjusted to pH 7.0 by 1.0 M H_2_SO_4_. One liter of lignin medium was prepared by adding 100 ml of 10 × M9, 10 ml of 12 g/L MgSO_4_·H_2_O, and 100 μl of trace element solution to make a certain lignin concentration. NH_4_Cl was used as nitrogen source, and the carbon to nitrogen ratio was adjusted with fermentation conditions. A limited glucose concentration of 5 g/L was added to lignin medium to support the cell growth. Seed cells were collected by centrifuging the seed culture at 4,000 rpm for 10 min and then incubated in the lignin medium. Fermentation was conducted at pH 7.0, 30°C, and 180 rpm in 250-ml Erlenmeyer flasks with a working volume of 50 ml.

### Measurement of Cell Growth and PHAs

As the lignin medium is a dark broth, the cell dry weight was determined to monitor the cell growth in fermentation with a gravimetric method. The fermentation broth containing the cells was centrifuged at 8,000 rpm for 10 min. The cell pellets were collected and washed twice with 0.9% sodium chloride solution, and then freeze-dried to a constant weight by a lyophilizer.

Polyhydroxyalkanoate content in dried cells was determined by a gas chromatography–mass spectrometer (GC–MS) method (Lin et al., 2016; Fra-Vazquez et al., 2019). Freeze-dried cells (20 mg) were loaded into a sealed stainless-steel chamber with a polytetrafluorethylene (PTFE) lining. A solution of 2 ml 15% (v/v) sulfuric acid in methanol, and 2 ml trichloromethane were mixed with the dried cells. The mixture was then subjected to methanolysis at 100°C for 4 h. After methanolysis, the sample was cooled to room temperature and moved into a 10 ml centrifuge tube with the addition of 1 ml ddH_2_O. The lower trichloromethane organic phase containing the resulting methyl esters was separated by centrifugation at 5,000 rpm for 5 min. The trichloromethane layer was filtered with a 0.22-μm PTFE membrane for GC–MS analysis.

The GC–MS system consists of SHIMADZU GC-2010 PLUS gas chromatography, a SHIMADZU GCMS-QP2020 mass spectrometer, and a fused-silica capillary column (30 m × 0.25 mm i.d., 0.25 μm DB-5MS, J & W Scientific, Folsom, United States). It was operated in constant linear velocity mode at 32.3 cm/s with a split ratio of 50:1. The temperatures of the interface and ion source were set to 270 and 230°C, respectively, while the inlet temperature was 260°C. The column temperature was initially held at 40°C for 5 min, ramped at 5°C/min to 250°C, and maintained for 20 min. All experiments were performed in triplicate.

### Characterizations of the Lignin Fractionated From Corn Stover

The linkages and hydroxyl groups of the fractionated lignin from corn stover biomass were analyzed using 2D NMR and ^31^P NMR (Liu et al., 2019a; Meng et al., 2019; Xu et al., 2022). The lignin molecular weight distribution before and after fermentation was analyzed by gel permeation chromatography (GPC). The analysis was carried out on a Waters e2695 system equipped with a variable 2489 UV/Vis detector at 280 and 254 nm and two tandem 30 cm × 7.8 mm TSKgel GMPWxl column (TOSOH, Tokyo). As alkaline solution can dissolve the lignin, 0.1 M NaOH was employed as a mobile phase with a flow rate of 1.0 ml/min (Liu et al., 2017; Xu et al., 2022).

### Analysis Methods

Composition analysis of corn stover and APL was conducted according to the laboratory analytical procedures (LAPs) of the National Renewable Energy Laboratory (NREL), Golden, CO, United States. The sugars were analyzed by an HPLC system equipped with a Waters 2414 RI detector and Bio-Rad HPX-87H column using 5 mM H_2_SO_4_ as a mobile phase with a flow rate of 0.6 ml/min at 65°C. Error bars in the tables and figures represent the SD of the replicates.

## Results and Discussion

### PHA Accumulation in *Pseudomonas putida* KTU Using an Actual Lignin Stream

**Figure 2:**
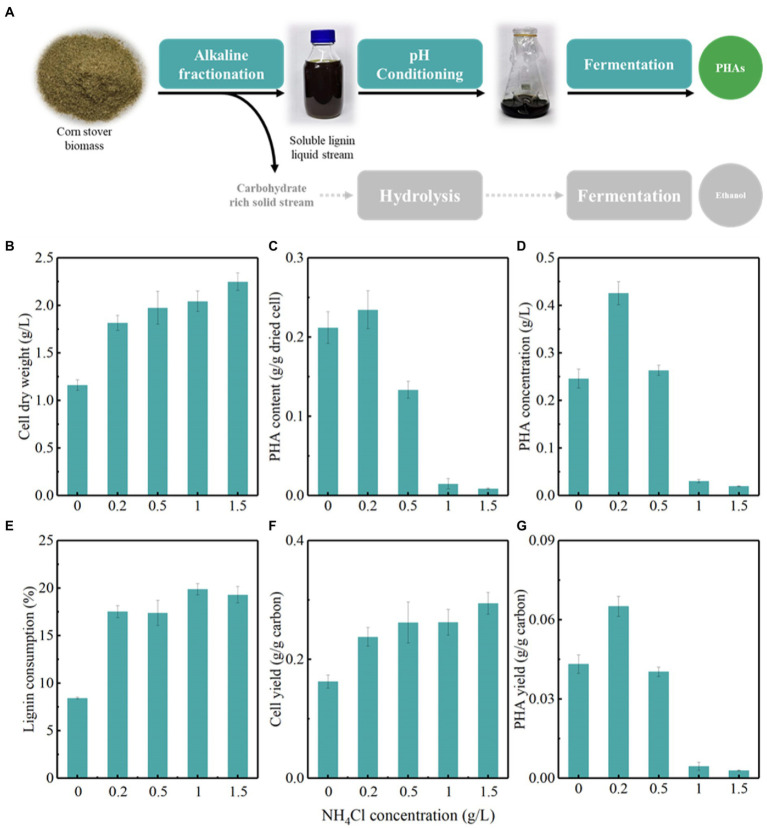
The optimization of nitrogen concentration for the accumulation of polyhydroxyalkanoates (PHAs) by *Pseudomonas putida* KTU grown on an actual lignin stream. **(A)** Represents the bioconversion of lignin to PHAs by integrating lignin fractionation, pH conditioning, and fermentation; **(B–G)** represents the production of PHAs using *P. putida* KTU grown on alkaline pretreated liquor (APL).

Microbial conversion of lignin to PHAs holds great promise for an economically competitive lignin valorization and environmental sustainability (Figure 1; Linger et al., 2014; Liu et al., 2019b). For bioconversion, lignin substrates should be water soluble and bioavailable for microbes. Herein, promising alkaline fractionation was employed in the present study to yield a soluble lignin stream, named APL, suitable for bioconversion (Table 1; Figure 2A). This soluble lignin was adjusted to neutral and used as carbon source by *P. putida* to produce PHAs.

*Pseudomonas putida* accumulates PHAs under nutrient imbalance conditions, such as excess carbon sources and limited nitrogen sources. It is thus necessary to assess the effects of the carbon to nitrogen ratio on the PHA-storing capacity of *P. putida* KTU (Figures 2B–G). The results showed that the PHA-storing capacity of *P. putida* KTU depended on the carbon to nitrogen ratio employed. At a certain lignin concentration, a lower carbon to nitrogen ratio promoted the cell growth of *P. putida* KTU, while a higher carbon to nitrogen ratio facilitated the PHA accumulation (Figures 2B,C). The cell dry weight increased from 1.2 to 2.3 g/L with NH_4_Cl concentration increasing from 0 to 1.5 g/L. Correspondingly, the lignin consumption increased from 8.4 to 19.3%, and a high nitrogen concentration facilitated the lignin consumption to support cell growth (Figures 2E,F). Interestingly, a higher PHA content of 0.23 g/g dried cells was obtained at NH_4_Cl concentration of 0.2 g/L (Figures 2C,G). Further increasing nitrogen concentration adversely affected PHA accumulation in *P. putida* KTU. As a result, the highest PHA concentration reached 0.43 g/L at the optimal NH_4_Cl concentration of 0.2 g/L (Figure 2D).

These results suggested that *P. putida* KTU can successfully synthesize PHAs using an actual lignin stream. Previous studies showed that using a 10 g/L lignin-enriched biorefinery residue, the engineered *P. putida* A514 can produce a PHA titer of 0.16 g/L under nitrogen-limiting conditions (Lin et al., 2016). When using lignin-derived aromatic compounds, engineered *P. putida* A514 can produce a PHA titer of 0.24 g/L from vanillic acid (Wang et al., 2018). *Pseudomonas putida* KT2440 can accumulate PHAs with a content of 0.32 g/g dried cells and a concentration of 0.25 g/L using APL as a sole carbon source (Linger et al., 2014), while engineered *P. putida* AG2162 can produce 0.24 and 0.12 g/L PHAs using *p*-coumaric acid and APL with a fed-batch fermentation, respectively (Salvachua et al., 2020a). In the present study, *P. putida* KTU showed excellent PHA-storing capacity, as it obtained a considerable PHA content and concentration from APL. Furthermore, the PHA-storing performance of *P. putida* KTU can be regulated by optimizing the carbon to nitrogen ratio. Previous studies reported that a higher PHA content in engineered *P. putida* A514 was obtained using starved nitrogen of 0.065 g/L NH_4_Cl from lignin-rich residue (Lin et al., 2016). Interestingly, engineered *P. putida* A514 produced a higher PHA concentration under NH_4_Cl concentration of 1 g/L from vanillic acid (Wang et al., 2018). Nutrient-rich conditions of 15 mM (NH_4_)_2_SO_4_ significantly promoted the cell growth of ligninolytic microbes and thus the lignin bioconversion compared with nitrogen-limiting conditions of 1 mM (NH_4_)_2_SO_4_, under which a higher PHA content was obtained (Salvachua et al., 2015). These results implied that limited nitrogen promoted the PHA accumulation, but the effects of nitrogen dose on PHA-storing performance could be contingent on the strains and carbon source employed.

Overall, *P. putida* KTU shows a promising capacity to grow on actual lignin medium for PHA accumulation. The PHA-storing performance is dependent on the carbon to nitrogen ratio employed.

### The Ligninolytic Capacity of Genome-Reduced *Pseudomonas putida*

**Figure 3:**
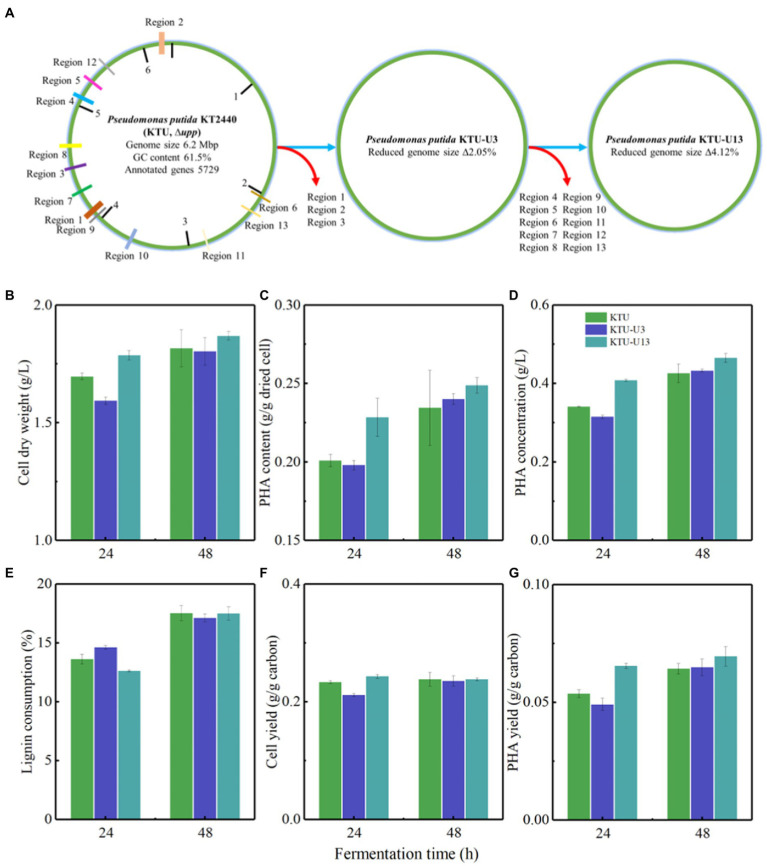
The ligninolytic capacity of genome-reduced *Pseudomonas putida* KTU, KTU-U3, and KTU-U13 grown on APL. **(A)** Represents the deleted regions of the genomic islands on the chromosome of *P. putida* KTU, KTU-U3, and KTU-U13. **(B–G)** Represents the production of PHAs using *P. putida* KTU, KTU-U3, and KTU-U13.

The ligninolytic capacity and PHA accumulation of genome-reduced *P. putida* strains was evaluated to understand the cell growth behaviors on APL medium and identify promising microbial chassis for synthetic biology applications (Figures 3A–G). Figure 3 shows the PHA fermentation performance using genome-reduced *P. putida* on APL medium. The results showed that genome-reduced *P. putida* KTU-U13 with the largest deletion of genomic islands had a higher cell dry weight at 24 and 48 h compared with original *P. putida* KTU and genome-reduced *P. putida* KTU-U3 (Figure 3B). Interestingly, KTU-U13 yielded PHA contents of 0.224 and 0.248 g/g dried cells at 24 and 48 h, respectively (Figure 3C). The corresponding PHA concentrations reached 0.42, 0.43, and 0.47 g/L for KTU, KTU-U3, and KTU-U13, respectively (Figure 3D). KTU-U13 accumulated 7.0% higher PHA content and 12% higher PHA concentration than KTU. These results suggested that KTU-U13 can effectively accumulate PHAs on APL.

Lignin types could determine its bioconversion performance and thus product synthesis. A water-soluble lignin, calcium lignosulfonate (CLS), was further used as carbon source for PHA production by genome-reduced *P. putida* strains. The results showed that the genome-reduced *P. putida* can grow on CLS medium and accumulate PHAs (Supplementary Figure 1). KTU-U13 grown on CLS generated a higher PHA content and concentration, followed by KTU-U3 and KTU, showing a similar trend as that on APL. However, the strains grown on CLS had lower cell dry weight, PHA content, and concentration compared with those on APL. The results indicated that the different lignin fractionation methods will yield different lignin derivatives and thus significantly affected the bioconversion performance. APL could be a preferred carbon source of genome-reduced *P. putida* compared with CLS.

**Figure 4:**
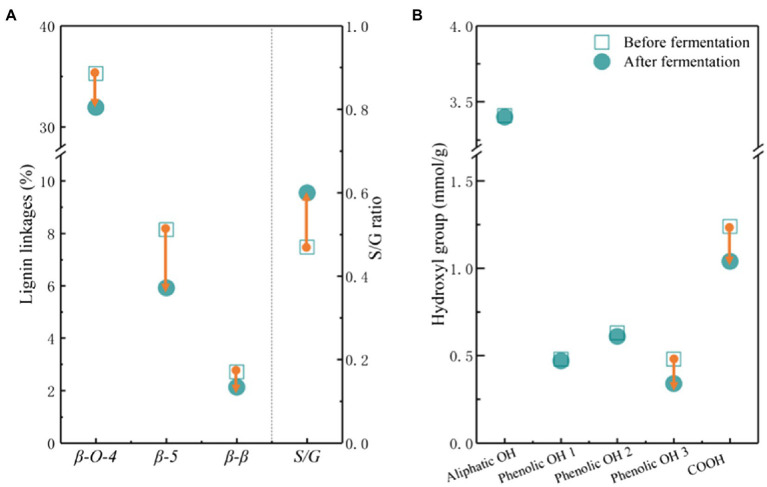
The linkages **(A)** and the hydroxyl groups **(B)** in lignin before and after fermentation using genome-reduced *Pseudomonas putida* KTU-U13. S/G ratio represents the ratio of syringyl and guaiacyl units. Phenolic OH 1 ~ 3 represents the phenolic OH of C5 substituted, guaiacyl, and *p*-hydroxyl phenyl, respectively.

The molecular weight distribution is one of the key factors affecting the bioavailability of lignin to microbes. APL possessed a lower number average molecular weight (*M*_n_) and a higher weight-average molecular weight (*M*_w_), and thus a higher dispersity (Đ) than CLS (Supplementary Table 1). The molecular weight of APL was much lower than that of other lignins, such as Kraft lignin, acid, and organosolv pretreated lignins, indicating the higher solubility and bioavailability of APL (Tolbert et al., 2014; Zhao et al., 2016). The *M*_n_, *M*_w_ and dispersity of APL and CLS were almost increased after fermentation possibly due to the synergistic effects of lignin degradation and utilization by ligninolytic *P. putida*. *P. putida* can not only secrete the extracellular enzymes to degrade the lignin, but also prefer to consume small molecules and leave large molecular fragments in fermentation broth (Liu et al., 2017; Salvachua et al., 2020c). The analysis of lignin linkages supported the above results as the major linkages of lignin were further degraded by ligninolytic *P. putida* during fermentation (Figure 4A; Supplementary Figure 2). Interestingly, the S/G ratio was increased after fermentation because ligninolytic *P. putida* preferred to consume G and H units (Notonier et al., 2021). After fermentation, the carboxyl group and *p*-hydroxyl phenyl OH group were decreased (Figure 4B), indicating the effective catabolism of more hydrophilic molecules. The results suggested that genome-reduced *P. putida* could selectively convert lignin fractions and lignin chemistries affected its bioconversion efficiency.

All above results suggested that the conversion performance of lignin depends on the ligninolytic capacity of the strains and the fractionated lignin types used. The genome-reduced *P. putida* KTU-U13 showed the good ligninolytic capacity on APL and the superior synthetic performance of PHAs, which could be used as a promising chassis for lignin bioconversion.

### pH Control Improved the PHA Titer in Genome-Reduced *Pseudomonas putida*

**Figure 5:**
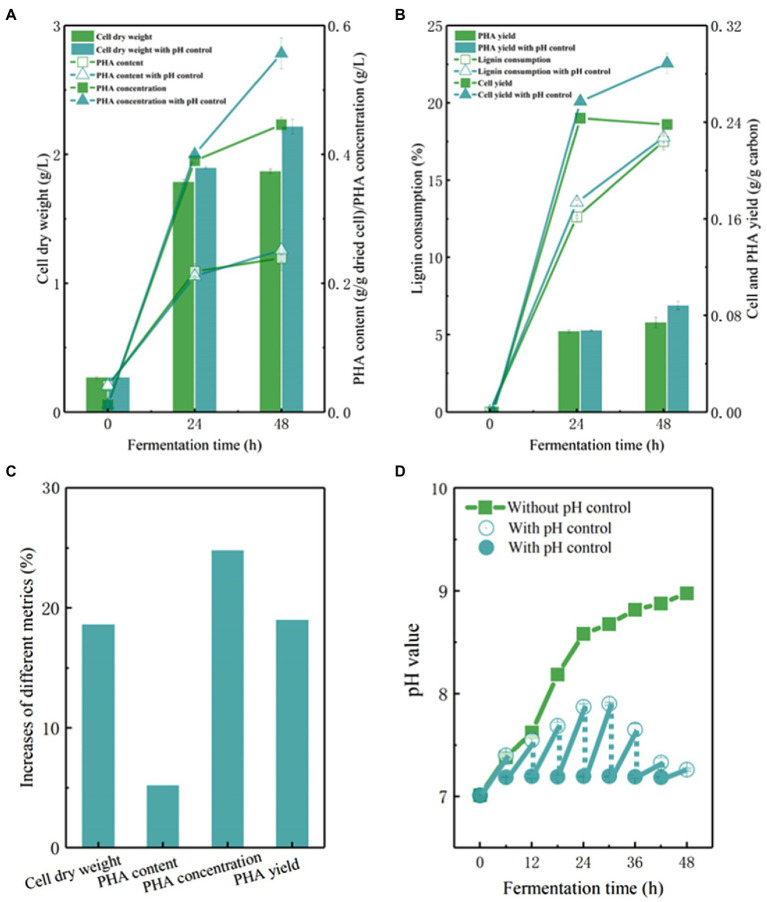
The pH control strategy of fermentation using genome-reduced *Pseudomonas putida* KTU-U13 grown on APL. **(A–D)** Represents the cell growth and PHA accumulation using P. putida KTU-U13 with pH control strategy.

The pH value of fermentation broth is one of most important environmental factors affecting fermentation efficiency (Figures 5A–D). The initial pH value of the APL medium was adjusted to 7.0, which is the optimal pH condition for *P. putida*. An interesting observation was that the pH value of APL medium was increased up to 9.0 with the progression of fermentation by KTU-U13 (Figure 5D). Previous studies also reported that the pH value reached to approximately 9.0 in the bioconversion of lignin or aromatic compounds by ligninolytic microbes, while the cell growth was accompanied by an increase in the pH value, showing a different trend as that using glucose as carbon source (Kosa and Ragauskas, 2012; Liu et al., 2018). An increased pH value in fermentation has also been observed in another study when lignin or aromatics were used as carbon sources (Zhang et al., 2021a). This phenomenon has not been explained clearly in microbial conversion of lignin-derived aromatics. The possible reason is that both aromatic degradation and PHA synthesis need a reducing equivalent (NADPH+H^+^), which are generated from the starting lignin-derived aromatic substrates (Borrero-de Acuña et al., 2014; Erickson et al., 2022).

As pH variation may affect the fermentation performance, a pH control strategy was designed by adjusting the pH value to approximately 7.0 every 6 h during fermentation (Figure 5D). As expected, the pH control strategy facilitated cell growth and improved fermentation performance. The cell dry weight increased to 2.25 g/L, representing a 19% increase (Figures 5A,C). The PHA concentration and yield increased by 23 and 19%, respectively, with pH control strategy (Figures 5B,C). These results suggested that the pH value affected the bioconversion of lignin to PHAs, and a pH control strategy is necessary to maintain a stable environment to facilitate lignin metabolism and PHA synthesis in genome-reduced *P. putida* KTU-U13.

### Evaluating the Harvesting Time of PHAs in Genome-Reduced *Pseudomonas putida*

As PHAs are synthesized and stored as carbon and energy sources by microbes, PHA granules could be metabolized by their own intracellular depolymerase to support the cell growth when the carbon source was depleted (Cai et al., 2009; Cha et al., 2020). On-site monitoring of lignin substrate is still difficult until now, and it is thus crucial to evaluate the balance point of PHA synthesis and degradation in fermentation.

Supplementary Figure 3 shows the fermentation kinetics of KTU-U13 grown on APL medium. Cell growth and PHA accumulation are a function of the growth phase during the fermentation. Cell growth was promoted on APL medium during the earlier stage of fermentation and reached 1.96 g/L at 36 h fermentation. The cell growth trend was consistent with the carbon utilization as lignin consumption was sharply increased to 16.5% at 36 h. However, the cell dry weight was obviously decreased after 48 h of fermentation, possibly due to the complete consumption of the small lignin molecules in APL. Similar trends of the cell growth were observed when aromatic compounds were used as carbon sources for PHA accumulation by *P. putida* (Salvachua et al., 2020b).

In addition, PHA accumulation in KTU-U13 was also enhanced during the earlier stage of fermentation and then decreased after 48 h of fermentation (Supplementary Figure 3). The results indicated that KTU-U13 could start to degrade the PHA granules to supply the carbon and energy for cellular metabolism when the available APL was depleted, which was also supported by the PHA yield results. The decrease in PHA content in KTU-U13 during the late stage of fermentation could also contribute to the decrease of cell dry weight and cell yield. Previous studies reported similar trends of PHA accumulation, as the PHA content increased at the earlier stage of fermentation and then decreased after 120 h of fermentation by *P. putida* KT2440 with APL as the only carbon source (Salvachua et al., 2015). As a result, the PHA concentration reached the highest value of 0.55 g/L at 36 h fermentation and then decreased significantly with the progression of fermentation. The 36-h fermentation seemed to be the best harvesting time of PHAs by genome-reduced *P. putida* KTU-U13 grown on APL medium.

Therefore, the results highlighted that the PHA accumulation was associated with the fermentation time, and the fermentation optimization is helpful to understand the best harvesting time for PHAs. PHA accumulation could also be dependent on the ligninolytic *P. putida* strains, carbon source, and fermentation conditions employed.

### PHA Fermentation With High Lignin Concentration by Genome-Reduced *Pseudomonas putida*

It is generally required to employ a high substrate concentration to enable a high product titer in fermentation, which can simplify the process, make the most efficient use of fermenters, and reduce the capital cost. Unlike glucose substrate, the fractionated lignin contained various aromatic derivatives, which possessed varied molecular weights and poor water solubility (Liu et al., 2018; Zhao et al., 2021). The handling process of fermentation using lignin substrate could thus be different from that using sugar. Therefore, it is necessary to understand the growth behaviors of *P. putida* on high lignin concentration medium.

The production of PHAs was conducted with different fermentation modes and lignin concentrations by genome-reduced *P. putida* KTU-U13 (Supplementary Table 2). Batch fermentation modes were conducted at 10, 20, and 40 g/L lignin, and the cell dry weight was increased with the increase of lignin concentration, indicating that KTU-U13 can grow on medium with a high lignin concentration (Supplementary Figure 4). Some nitrogen sources could exist in APL as they are generated from corn stover in the fractionation. The nitrogen source content was increased at high lignin concentrations to facilitate cell growth (Liu et al., 2013). Unsatisfactorily, the PHA content and concentration decreased with increasing lignin concentration. The possible reasons for this phenomenon were that increases in lignin concentration may change the viscosity and the rheology behavior of the medium, as 40 g/L lignin used in the present study corresponded to more than 130 g/L total substrates in APL medium (Table 1). A highly viscous medium may adversely affect the dissolution and diffusion of oxygen, hindering lignin metabolism and PHA synthesis (Liu et al., 2018). In addition, aromatic derivatives possessing aldehyde and methoxy groups may enhance the inhibitory effects on the *P. putida* cells and lead to poor PHA accumulation (Shin et al., 2019; Ramirez-Morales et al., 2021). Furthermore, the salt concentration in APL from alkaline fractionation was increased and could be harmful to cell growth and PHA accumulation. These synergistic effects could adversely affect the PHA accumulation and restrain the improvement in PHA titer at high lignin concentrations.

Fed-batch strategies were designed to facilitate the PHA accumulation at high lignin concentrations (Supplementary Table 2). Fed-batch modes 1 and 2 were carried out with an initial lignin concentration of 20 g/L for 24 h fermentation, and the lignin medium was then changed by adding 20 g/L lignin solid or refreshing 20 g/L lignin medium at 24 h, respectively (Supplementary Figure 4). Although the cell dry weight increased in fed-batch modes 1 and 2, the PHA accumulation and concentration were still lower than those in batch modes 1 and 2 (Supplementary Figure 4). The results suggested that the obstacles relevant to high lignin concentrations were not eliminated in fermentation.

To improve the tolerance capacity of genome-reduced *P. putida*, the fermentation strategy with different incubation doses was further evaluated (Supplementary Table 2; Supplementary Figure 5). Interestingly, lignin consumption reached up to 17.2% at high incubation doses, contributing to the increase in cell dry weight. Higher PHA contents and concentrations were obtained with the incubation dose of OD 2 at 20 g/L lignin. The results highlighted that under a high lignin concentration; increases in incubation dose could enhance the tolerance capacity of cells to lignin and improve lignin consumption for PHA accumulation by genome-reduced *P. putida*.

### Fed-Batch Strategies Improved PHA Titers by Genome-Reduced *Pseudomonas putida*

**Figure 6:**
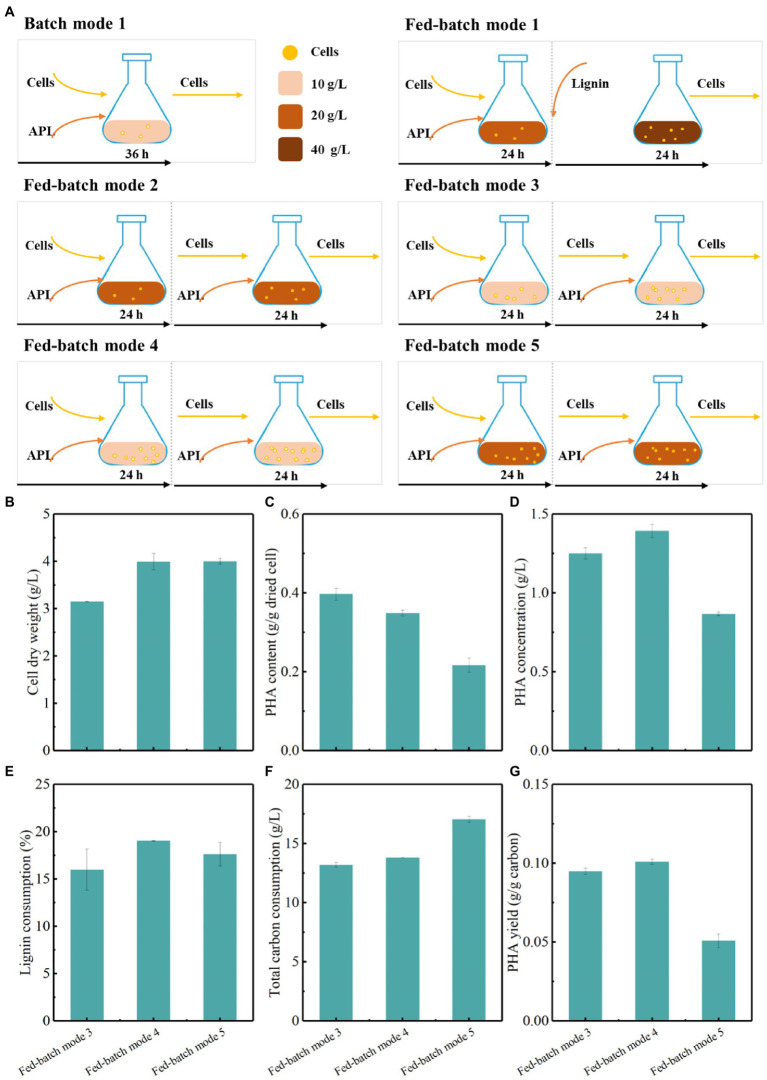
Fed-batch fermentation strategy for the production of PHAs by genome-reduced *Pseudomonas putida* KTU-U13 from APL. **(A)** Represents the fed-batch strategy used in fermentation; **(B–G)** Represents the fermentation results by *P. putida* KTU-U13.

To improve the PHA fermentation performance, other fed-batch strategies were further evaluated (Figure 6A; Supplementary Table 2). Fed-batch mode 3 was conducted at an initial lignin concentration of 10 g/L for 24 h fermentation and the lignin medium were refreshed with a lignin concentration of 10 g/L for another 24 h fermentation (Figure 6A). Compared with batch mode 1, fed-batch mode 3 obviously increased the cell dry weight, PHA content, and concentration, corresponding to 3.1 g/L, 0.4 g/g dried cells, and 1.2 g/L, respectively (Figures 6B–D). Meanwhile, lignin consumption reached up to more than 16%, and the PHA yield was 0.094 g/g carbon by genome-reduced *P. putida* KTU-U13 (Figures 6E–G). Compared with fed-batch mode 3, fed-batch mode 4 with a higher initial incubation dose produced the highest cell dry weight, PHA content and concentration of 4.0 g/L, 0.35 g/g dried cell, and 1.4 g/L, respectively. Fed-batch mode 4 increased the PHA titer by 14.3%, while it obtained higher lignin consumption and PHA yield than fed-batch mode 3. Fed-batch mode 5 was carried out at an initial lignin concentration of 20 g/L and a higher initial incubation dose for 24 h fermentation, and the lignin medium was then refreshed for another 24 h fermentation. Fed-batch mode 5 enhanced cell growth and PHA accumulation as compared with batch mode 2 at 20 g/L lignin (Figure 6). Although fed-batch mode 5 obtained a similar cell dry weight to fed-batch mode 4, the PHA accumulation and concentration were not further improved possibly due to the substrate effect of increased lignin concentration. The results highlighted that the fed-batch mode 4 was an implementable process that can reduce the inhibitory effects of lignin substrates, improve cell growth and PHA accumulation, and thus facilitate the improvement in lignin bioconversion by KTU-U13.

Overall, the bioconversion potential of the actual lignin stream was evaluated to produce PHAs by genome-reduced *P. putida*. The results highlighted that genome-reduced *P. putida* KTU-U13 showed the good ligninolytic capacity and PHA accumulation efficiency, which could act as an optimal chassis for the cell factory construction of PHA synthesis. Based on the origin of lignin, the optimization of fermentation strategies obviously improved the PHA titer, providing the promising approaches for lignin bioconversion.

## Conclusion

The bioconversion of lignin to PHAs was successfully achieved by genome-reduced *P. putida*. The genome-reduced *P. putida* exhibited ligninolytic capacity and PHA accumulation. *P. putida* preferred to consume small and hydrophilic lignin molecules to boost cell growth and PHA synthesis. The fermentation options promoted lignin bioconversion, cell growth, and thus PHA accumulation in genome-reduced *P. putida*. The fed-batch strategy improved lignin bioconversion and PHA accumulation at a high lignin concentration. Therefore, genome-reduced *P. putida* could be an optimal chassis for synthetic biology applications to facilitate lignin bioconversion and bioplastic production.

## Data Availability Statement

The original contributions presented in the study are included in the article/**Supplementary Material**; further inquiries can be directed to the corresponding author.

## Author Contributions

Q-JZ performed the experiments and data analysis and prepared the manuscript. TX conducted the characterization of lignin. HL, LX, and R-KZ contributed to lignin content analysis and fermentation data collection. B-ZL and Z-HL guided experiment design and the writing and editing of this manuscript. All authors contributed to the article and approved the submitted version.

## Funding

The work was financially supported by the Tianjin Fund for Distinguished Young Scholars (19JCJQJC63300) and the National Natural Science Foundation of China (21622605).

## Conflict of Interest

The authors declare that the research was conducted in the absence of any commercial or financial relationships that could be construed as a potential conflict of interest.

## Publisher’s Note

All claims expressed in this article are solely those of the authors and do not necessarily represent those of their affiliated organizations, or those of the publisher, the editors and the reviewers. Any product that may be evaluated in this article, or claim that may be made by its manufacturer, is not guaranteed or endorsed by the publisher.
