# Supplementary material for: Microbial Valorization of Lignin to Bioplastic by Genome-Reduced Pseudomonas putida
Source: Front Microbiol. 2022 May 30;13:923664. doi: 10.3389/fmicb.2022.923664 (PMC9189415; doi:10.3389/fmicb.2022.923664)
Supplement: Supplementary file 1 [file Data_Sheet_1.docx]

Supplementary Material

**Supplementary Table 1** Molecular weight distributions of the soluble lignins before and after fermentation using genome-reduced *P. putida* KTU-U13

| Samples | Before fermentation | | | After fermentation | | |
| --- | --- | --- | --- | --- | --- | --- |
|  | M_n_ | M_w_ | Dispersity | M_n_ | M_w_ | Dispersity |
| APL | 918 | 2003 | 2.2 | 837 | 2168 | 2.6 |
| CLS | 1004 | 1667 | 1.7 | 1106 | 1963 | 1.8 |

APL represents alkaline pretreated liquor; CLS represents calcium lignosulfonate; M_n_ represents number average molecular weight; M_w_ represents weight-average molecular weight.

**Supplementary Table 2** Fed-batch fermentation strategy to improve the production of polyhydroxyalkanoates (PHAs) by genome-reduced *P. putida* KTU-U13

| Modes | Lignin (g/L) | The strategies of refreshed lignin medium | Initial OD_600_ |  |
| --- | --- | --- | --- | --- |
| Batch mode 1 | 10 | Initial lignin concentration is 10 g/L for 36 h fermentation | 1 |  |
| Batch mode 2 | 20 | Initial lignin concentration is 20 g/L for 36 h fermentation | 1 |  |
| Batch mode 3 | 40 | Initial lignin concentration is 40 g/L for 48 h fermentation | 1 |  |
| Batch mode 4 | 20 | Initial lignin concentration is 20 g/L for 36 h fermentation | 2 |  |
| Batch mode 5 | 20 | Initial lignin concentration is 20 g/L for 36 h fermentation | 5 |  |
| Fed-batch mode 1 | 20 (0 h) +20 (24 h) | Initial lignin concentration is 20 g/L for 24 h fermentation, and lignin solid was added to make a final lignin concentration of 40 g/L for another 24 h fermentation | 1 |  |
| Fed-batch mode 2 | 20 (0 h) +20 (24 h) | Initial lignin concentration is 20 g/L for 24 h fermentation, the cells were collected into a new media with 20 g/L lignin for another 24 h fermentation | 1 |  |
| Fed-batch mode 3 | 10 (0 h) +10 (24 h) | Initial lignin concentration is 10 g/L for 24 h fermentation, the cells were collected into a new media with 10 g/L lignin for another 24 h fermentation | 2 |  |
| Fed-batch mode 4 | 10 (0 h) +10 (24 h) | Initial lignin concentration is 10 g/L for 24 h fermentation, the cells were collected into a new media with 10 g/L lignin for another 24 h fermentation | 5 |  |
| Fed-batch mode 5 | 20 (0 h) +20 (24 h) | Initial lignin concentration is 20 g/L for 24 h fermentation, the cells were collected into a new media with 20 g/L lignin for another 24 h fermentation | 5 |  |


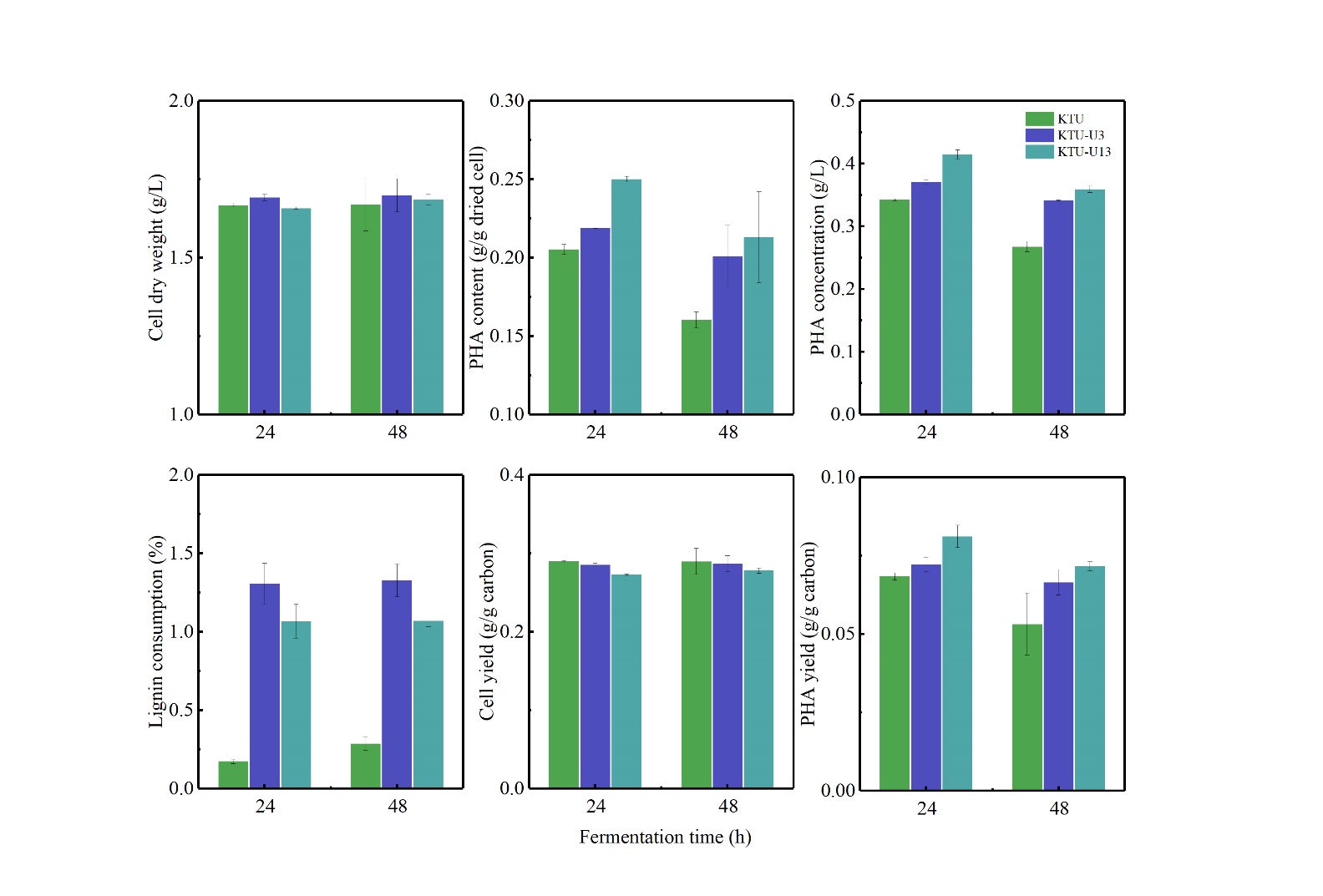


**Supplementary Figure 1** The ligninolytic capacity and polyhydroxyalkanoate accumulation of genome-reduced *P. putida* KTU, KTU-U3, KTU-U13 grown on calcium lignosulfonate (CLS)


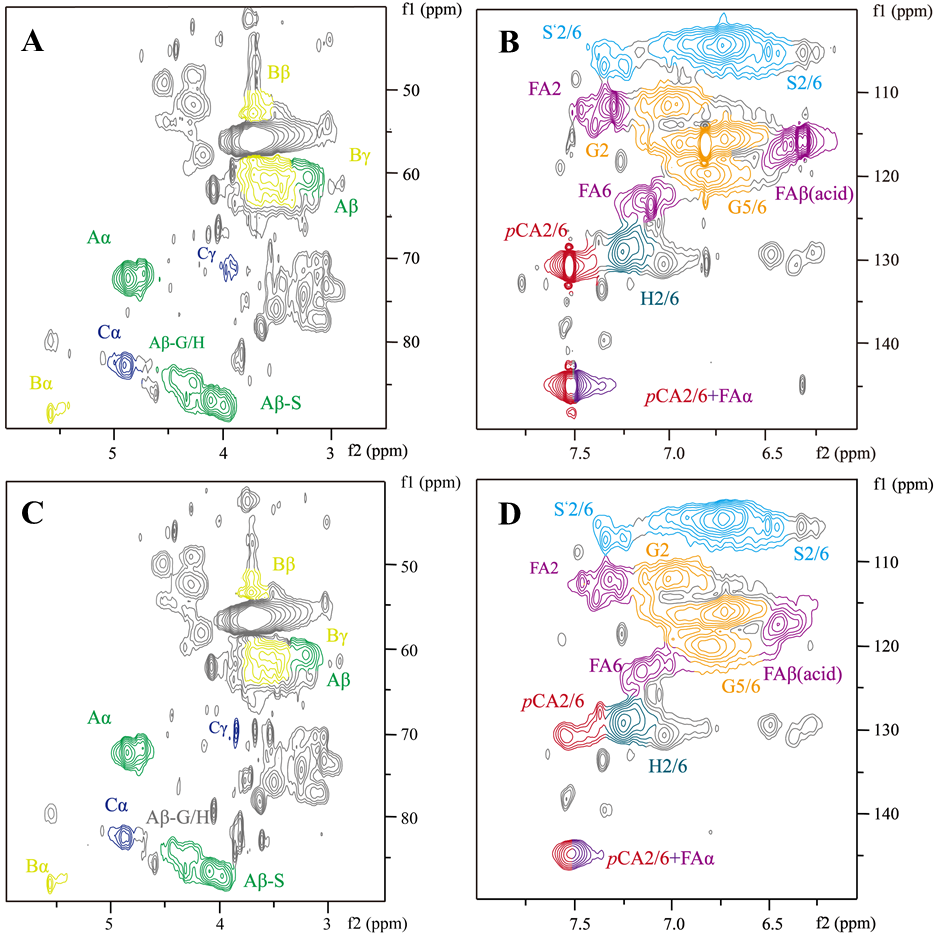


**Supplementary Figure 2** 2D NMR spectra from the fractionated lignin by alkaline method (A and B) and the lignin substrate after fermentation (C and D) using genome-reduced *P. putida* KTU-U13

**
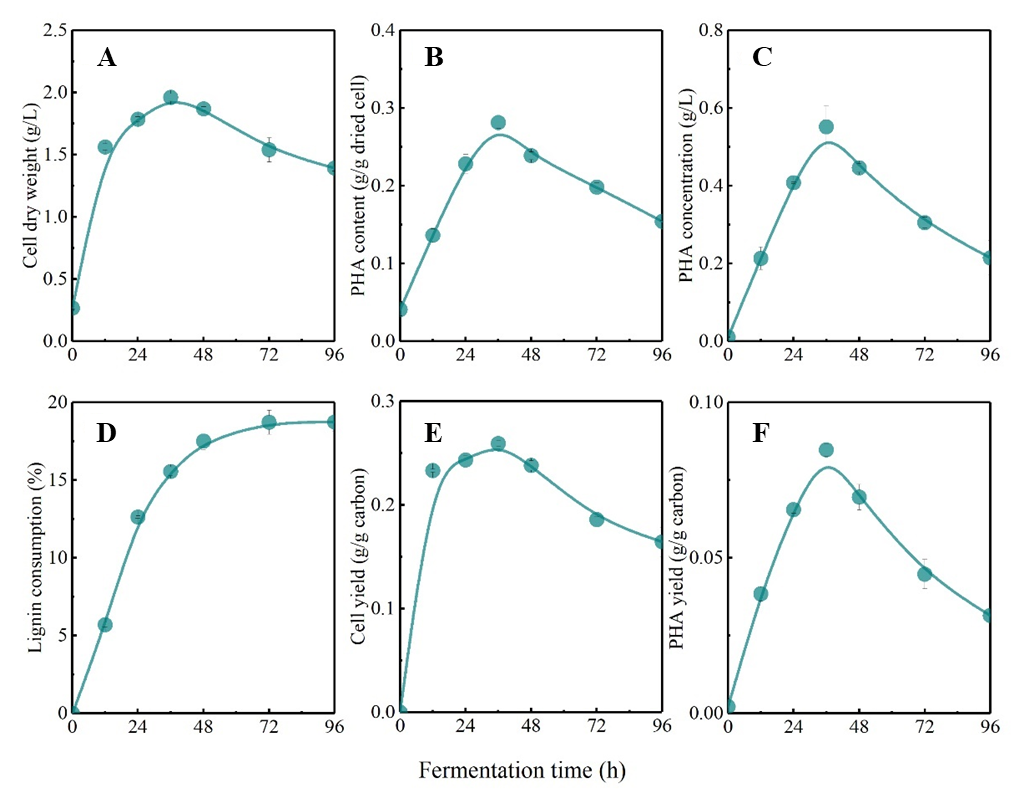
**

**Supplementary Figure 3** The harvesting time evaluation for the production of polyhydroxyalkanoates (PHAs) using genome-reduced *P. putida* KTU-U13 grown on alkaline pretreated liquor (APL).


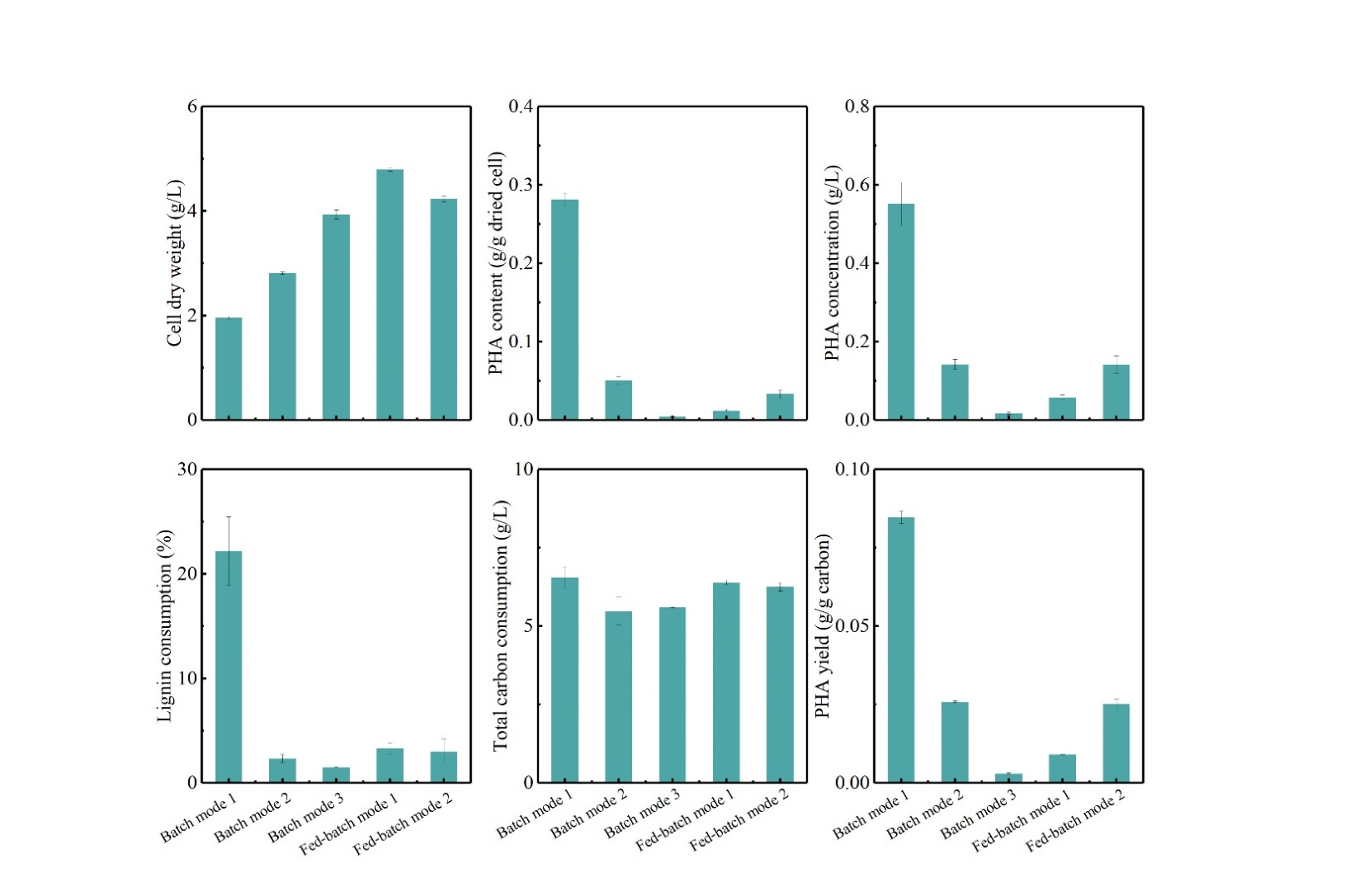


**Supplementary Figure 4** The production of polyhydroxyalkanoates (PHAs) at high lignin concentration in batch and fed-batch fermentation using genome-reduced *P. putida* KTU-U13


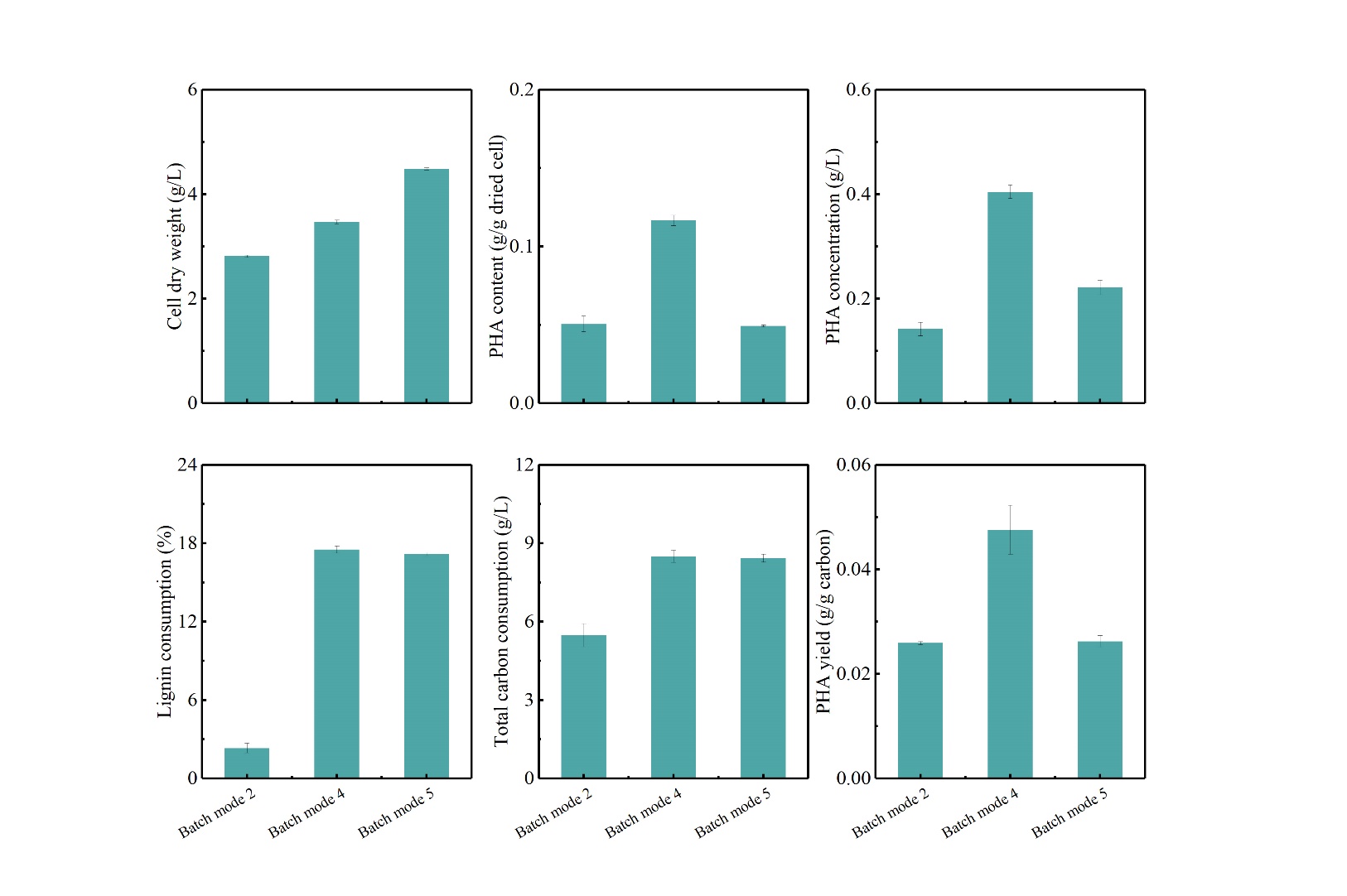


**Supplementary Figure 5** The production of polyhydroxyalkanoates (PHAs) at high lignin concentration in batch and fed-batch fermentation using genome-reduced *P. putida* KTU-U13
